# A Study of the Buffer Capacity of Polyelectrolyte Microcapsules Depending on Their Concentration and the Number of Layers of the Polyelectrolyte Shell

**DOI:** 10.3390/ijms23179917

**Published:** 2022-08-31

**Authors:** Egor V. Musin, Alexey V. Dubrovskii, Aleksandr L. Kim, Sergey A. Tikhonenko

**Affiliations:** Institute of Theoretical and Experimental Biophysics Russian Academy of Science, Institutskaya St., 3, 142290 Puschino, Moscow Region, Russia

**Keywords:** polyelectrolyte microcapsules, microcapsules, buffer capacity, polyelectrolyte layers, PMC, microcapsules concentration

## Abstract

Polyelectrolyte microcapsules are used in the development of new forms of targeted delivery systems, self-healing materials, sensors, and smart materials. Nevertheless, their buffer capacity has not been practically studied, although that characteristic makes it possible to estimate the change in the state of protonation of the entire polyelectrolyte system. This is necessary both for creating a buffer barrier system for pH-sensitive compounds (metals, enzymes, polyelectrolytes, drugs) and for the correct interpretation of the results of research and studying of the PMC structure. The buffer capacity of a PMC can be affected by the concentration of microcapsules in solution and the number of shell layers since the listed parameters affect other physicochemical properties of the PMC shell. This includes, for example, the electrical conductivity, permeability (of ions), osmotic pressure, charge density, etc. In this regard, we studied the change in the buffer capacity of polyelectrolyte microcapsules depending on their concentration and the number of shell layers. As a result, it was found that with an increasing concentration of microcapsules, the buffering capacity of the PMC increases, but at the same time, in the pH range from 4 to 5.5, the calculated buffering capacity of 1 billion capsules decreases with increasing their concentration. This effect may be associated with a decrease in the available -NH_2_ groups of the PMC’s shell. In addition, it was found that the main contribution to the buffer capacity of a PMC is made by the entire shell of the microcapsule and not just its surface. At the same time, the buffer capacity of the capsules has non-linear growth with an increase in the number of PMC shell layers. It is presumably associated either with a decrease in the polyelectrolyte layer with an increase in their number or with a decrease in the permeability of hydrogen protons.

## 1. Introduction

A solution’s buffer capacity (BC) is the ability to maintain a constant pH value when an acid or alkali is added [1]. The buffer capacity is typical for weak polyelectrolytes such as polyallylamine. Polyelectrolytes and their complexes can change the degree of their (de) protonation and charge density when the pH of the solution changes. A polyelectrolyte microcapsule shell consisting of polyelectrolyte complex will have buffer capacity, too [2].

Polyelectrolyte microcapsules (PMC) are supramolecular systems created by the layer-by-layer method [3]. The study of the buffer capacity of microcapsules makes it possible to estimate the change in the state of protonation of the entire polyelectrolyte system, which changes after both interpolyelectrolyte interactions inside the capsule and the interaction between the encapsulated substance and the shell polyelectrolytes. This parameter is necessary both for creating a buffer barrier for the surface, solution, and encapsulated pH-sensitive compounds (metals, enzymes, polyelectrolytes, drugs) and for the correct interpretation of the results of research. For example, the articles of Antipina, M.N. et al. [4] and Kazakova, L.I. et al. [5] demonstrated the diagnostic systems based on encapsulated pH-sensitive fluorescent labels (Amplex Red and SNARF-1). These dyes can change their fluorescence depending on the pH of the solution, but the presence of the buffer capacity of the polyelectrolyte microcapsule can distort the received signals. Additionally, the protonation level may influence the release rate of encapsulated proteins and the degradation of the PMC’s shell [6]. The proximity of pH 7 explains the increase in protein release and dissociation of PAH with increasing pH to the point of charge exchange of the amino group of polyelectrolytes as a result of the dissociation of the microcapsule. This effect is associated with the electrostatic nature of the polyelectrolyte-protein and polyelectrolyte-polyelectrolyte interactions. At the same time, Valentin V. Lulevich and Olga I. Vinogradova demonstrated the softening of the capsules at a high pH. It is related to a decrease in the charge density of a polycation, which leads to a reduction in the number of ionic cross-links. In acidic solutions, the capsules demonstrate low stiffness, which is mostly connected with the increased permeability of the polyelectrolyte shell [7,8].

Dubrovsky et al. demonstrated the buffer capacity of polyelectrolyte microcapsules and the dependence of the behavior of the buffer capacity on the presence of an encapsulated protein [2], the ionic strength, and the temperature of the solution [9]. Additionally, these works confirmed that the buffer properties of microcapsules are determined by polyallylamine (PAH) sites uncompensated with polystyrene sulfonate in the composition of a PMC’s shell.

We assume that the number of layers of the polyelectrolyte shell of the microcapsule may be one of the possible reasons for the change in the buffer capacity of PMC. In particular, the ion permeability of a polyelectrolyte shell may be reduced by increasing the number of polyelectrolyte layers of it. For example, A.I. Petrov et al. have been studying the rate of dissolving the naked magnesium oxalate microcrystals and the microcrystals coated with multilayered polyelectrolyte shells of molecular composition (polyallylamine/polystyrene sulfonate)n ((PAH-PSS)n) and (polyallylamine/polystyrene sulfonate)n(polyallylamine) ((PAH-PSS)nPAH)). It was shown that the kinetics of the release of magnesium ions into solution decreased with the number of layer shells *n* > 10 [10]. Possible factors that control ion permeability are the charge density of the polyelectrolyte complex that forms the PMC shell and the number and size of its hydrophobic regions [11,12,13]. Thus, an increase in the number of layers of the PMC shell can decrease the permeability of other ions, for example, H^+^ and OH^−^ [14], and, consequently, change the behavior of the buffer capacity of the PMC at different pH.

Another confirmation of the possible influence of the number of microcapsule shell layers on its buffer capacity is the change in its electrical conductivity. In the work of Tao Sun et al. [15], it was shown that the conductivity of microcapsules (PAH/PSS)_6_ and (PAH/PSS)_9_ differ from each other. In turn, the electrical conductivity of the polyelectrolyte complex of the PMC shell depends on the density of ionogenic groups and ionic and nonionic intra- and intermolecular interactions of individual monomers. It depends on the level of protonation and solvation of polyions [16]. Thus, the buffer capacity of PMC may vary with the number of PMC layers since the BC of polyelectrolytes and their complexes depend on the same parameters as the electrical conductivity of polyelectrolytes described above [17,18,19,20].

Additionally, the buffer capacity of PMC may depend on the even/odd number of polyelectrolyte layers in their shell. The work of Halozan et al. showed that any changes in the state of electrical neutrality of PMC lead to a change in the concentration of Na^+^ ions inside microcapsules and to a change in the concentration of [H^+^] in the internal solution, which is associated with a change in the level of protonation of polyelectrolytes [21]. Thus, the even/odd number of PMC polyelectrolyte layers may change its buffer capacity due to the deviation of local ion concentrations and pH [22].

The concentration of polyelectrolyte microcapsules may also be one of the reasons for the change in the buffer capacity of polyelectrolyte microcapsules. Qiyun Tang and Alan R. Denton found that changing the number of microcapsules leads to a non-linear pH shift, which is associated with a non-linear change in the ion densities inside the cavities of the PMCs [23]. In addition, as the volume fraction of particles increases, the so-called “clustering” effect occurs [24]. An increase in particles prevents the fluid from moving around them, leading to higher viscosities [25,26]. At the same time, accumulated particles with different effective volumes (different sizes) may lead to fluid stagnation inside and between particles, leading to a rapid decrease in the fluidity of the suspension [27]. In turn, it may change the local concentration of [H^+^] and the buffer capacity of the entire system.

The number of capsules and the number of layers of the PMC shell are key parameters in developing targeted delivery systems, self-healing materials, sensors, and smart materials [22,24,28]. In this case, as described above, various properties of microcapsules (permeability, electrical conductivity, etc.) may depend on the concentration of microcapsules and the number of layers of their shell. This may also indicate a possible dependence of the buffer capacity of polyelectrolyte microcapsules on these conditions. Thus, it is necessary to study the buffer capacity of polyelectrolyte microcapsules depending on their concentration and the number of shell layers. Thus, our work aims to study the buffer capacity of various types of polyelectrolyte microcapsules, depending on the concentration of microcapsules in solution and the number of layers of their shell.

## 2. Results and Discussion

In previous works, we revealed the presence of a buffer capacity (BC) in polyelectrolyte microcapsules consisting of PAH and PSS. Additionally, we found the dependence of the buffer capacity’s behavior on the PMC shell’s composition, the presence of an encapsulated protein, the magnitude of ionic strength, and the temperature of the solution. These studies demonstrated that the buffer properties of microcapsules are determined by the charged regions of PAH (uncompensated with PSS) in the composition of their shell [2,9]. However, as described above, the number and density of PAH charged groups (distance between nearest groups of -NH_3_^+^ groups) also depend on the number of microcapsule shell layers and the number of microcapsules themselves. Thus, the parameters described above can also affect the behavior of the PMC buffer capacity.

### 2.1. Investigation of the Buffer Capacity of PMC Depending on Their Concentration

**Figure 1:**
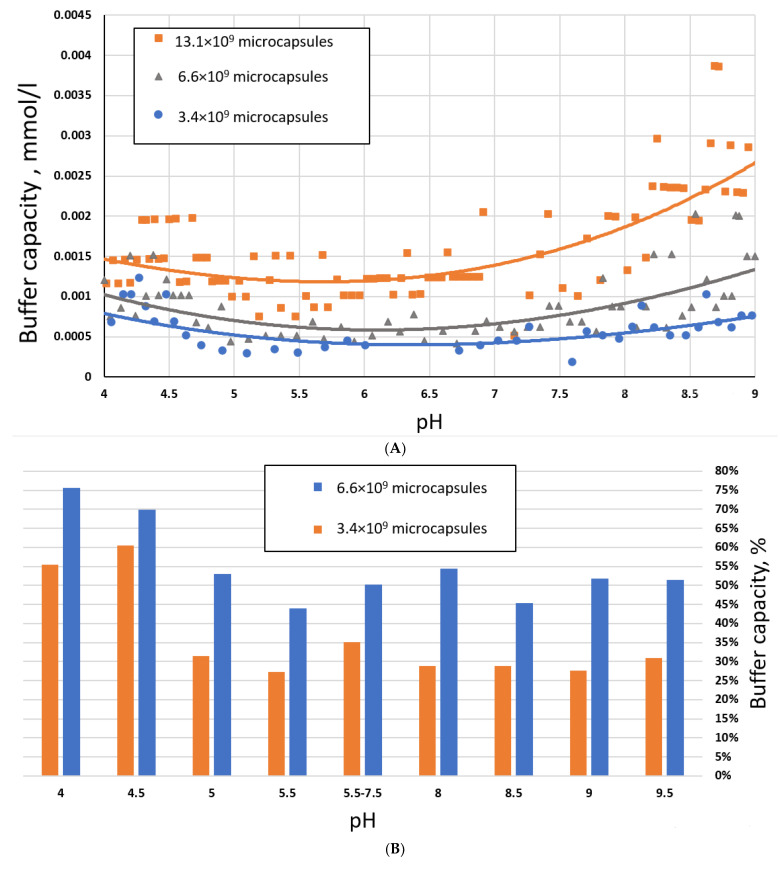

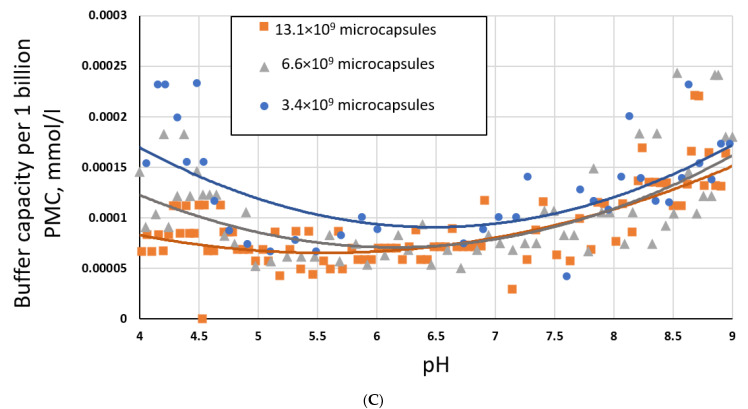
Buffer capacity of PMC composition (PSS/PAH)_3_ in the pH range from 4 to 9 depending on the concentration of microcapsules. (**A**) Buffer capacity of PMC depending on the concentration of microcapsules. (**B**) Relative buffer capacity of PMC, where 100% is buffer capacity of 13.1 × 10^9^ microcapsules. (**C**) The calculated buffer capacity per 1 billion microcapsules.

We have studied BC with a different titer of polyelectrolyte microcapsules: 3.4 × 10^9^ microcapsules, 6.6 × 10^9^ microcapsules, and 13.1 × 10^9^ microcapsules. The results of this study are presented in Figure 1.

As can be seen from Figure 1A, the buffer capacity of the entire suspension increases with an increase in the concentration of microcapsules, primarily due to an increase in the amount of polyelectrolyte in the system with an increase in the concentration of PMC. In the pH range from 5.5 to 9, we observe a proportional increase in the buffer capacity of the suspension of capsules (Figure 1B). While in the pH range from 4 to 5.5, the dependence has a different character (Figure 1B). As seen from Figure 1C, the calculated buffer capacity per 1 billion capsules in the pH range from 4 to 5.5 decreases after increases in PMC concentration. An increase in PMC concentration may lead to the growth of the charge density of the capsule shell [23] by reducing the distance between the unpaired sites of PAH. This, in turn, leads to a decrease in the effective hydrodynamic radius of PAH and an increase in hydrophobic and hydrogen interactions. As a result, the solubility and the number of -NH_2_ amino groups of PAH decrease, capable of acting as [H^+^] acceptors. However, at the same time, the number of donor -NH_3_^+^ groups does not decrease [18,19,29] because charged NH_3_^+^ groups were hydrated after water rearrangement between the approaching charges [30].

### 2.2. Study of the Buffer Capacity of PMC Depending on the Number of Layers of the Polyelectrolyte Shell

**Figure 2:**
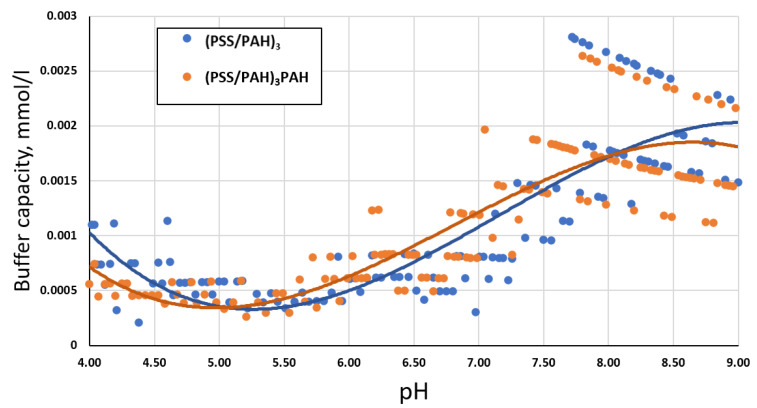
Buffer capacity of PMC in the pH range from 4 to 9 with shell compositions (PSS/PAH)_3_ and (PSS/PAH)_3_PSS.

The next stage of the research is the study of PMC’s buffer capacity with different shell compositions: (PSS/PAH)_3_ and (PSS/PAH)_3_PSS. The results obtained are shown in Figure 2.

As seen in Figure 2, the presence of an additional PSS layer did not affect the change in the buffer capacity of PMC. Presumably, this effect may be due to the fact that the entire volume of PAH in the shell of the polyelectrolyte microcapsule is responsible for the buffer capacity and not just its surface, and its amount in both samples did not change.

**Figure 3:**
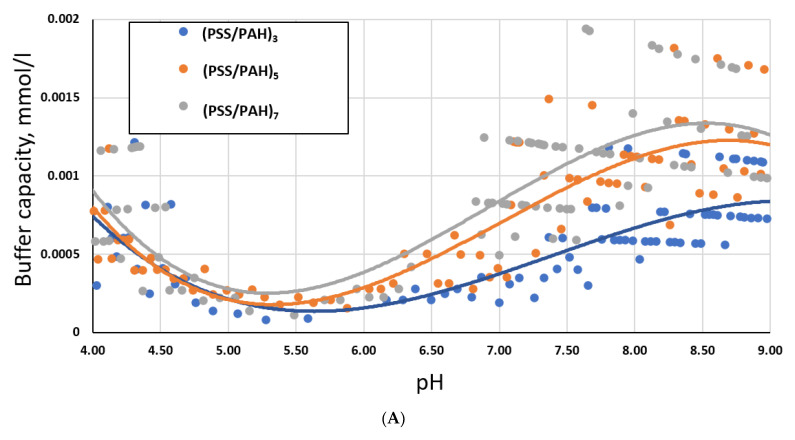

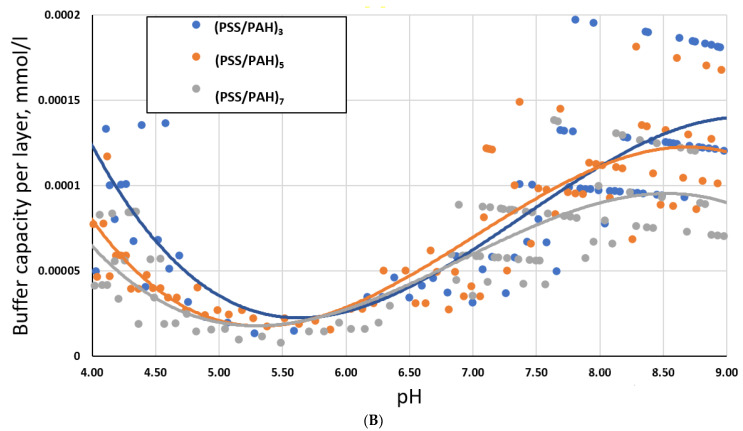
Buffer capacity of PMC in the pH range from 4 to 9 with shell compositions: (PSS/PAH)_3_, (PSS/PAH)_5_, (PSS/PAH)_7_. (**A**) Buffer capacity of PMCs. (**B**) Average buffer capacity of PMCs per 1 layer.

If the above assumption is correct, then the increased number of layers of PMC will increase the buffer capacity. To confirm this hypothesis, we measured the buffer capacity of a suspension of microcapsules with shell compositions (PSS/PAH)_3_, (PSS/PAH)_5_, and (PSS/PAH)_7_. Figure 3 shows the results obtained.

As can be seen from the results obtained (Figure 3A), with an increase in the number of PMC shell layers, their buffer capacity also increases, which confirms the hypothesis described above. However, at the same time, this increase is not proportional. For illustration purposes, the buffer capacity of one layer of microcapsules was calculated by dividing the buffer capacity of the PMC sample by the number of their layers. The obtained results are demonstrated in Figure 3B. The figure shows that the buffer capacity of one layer decreased with an increase in the number of layers. This effect may be due to several reasons. First, at the preparation stage of PMC, the amount of adsorbed polyelectrolyte layers decreases with increasing layer number [31]. As a result, the amount of PAH amino groups available for (de)protonation decreases. Secondly, with an increase in the number of layers, the permeability of the PMC shell for hydrogen protons [10,14] may decrease due to an increase in the charge density of the shell (distance decreases between nearest groups of -NH_3_^+^ groups) and the number of hydrophobic regions. As a result, it leads to a decrease in the buffer capacity of the microcapsule calculated per 1 layer.

## 3. Materials and Methods

### 3.1. Materials

The polyelectrolytes polystyrene sulfonate sodium (PSS) and polyallylamine hydrochloride (PAH) with a molecular mass of 70 kDa, as well as ethylenediaminetetraacetic acid disodium salt dihydrate (EDTA), were purchased from Sigma (St. Louis, MO, USA). Sodium chloride, sodium sulfate, sodium carbonate, and calcium chloride were obtained from “Reahim”.

### 3.2. Preparation of CaCO_3_ Microspherulites

At a stirring of 0.33 M Na_2_CO_3,_ the 0.33 M CaCl_2_ was added. The stirring time was 30 s. The suspension was maintained until complete precipitation of the formed particles. The process of “ripening” of microspherolites was controlled with the help of a light microscope. Then, the supernatant was decanted, and the precipitate was washed with water and used to prepare PMC. The microparticles were obtained with an average diameter of 4.5 ± 1 μm.

### 3.3. Preparation of Polyelectrolyte Microcapsules

The polyelectrolyte microcapsules were obtained by layer-by-layer adsorbing the negatively or positively charged polyelectrolytes onto CaCO_3_ microspherulites, followed by the dissolution of CaCO_3_. At the moment of dissolution of the CaCO_3_ core, the inner space of PMC is filled by an interpolyelectrolyte complex [5]. Layer-by-layer adsorption of PAH and PSS on the CaCO_3_ microspherulites surface was carried out in the polyelectrolytes solutions (concentration 2 mg/mL + 0.5 M NaCl). After each adsorption, the CaCO_3_ particles with adsorbed polyelectrolytes were triple-washed with a 0.5 M NaCl solution, which was necessary to remove unadsorbed polymer molecules. The particles were separated from the supernatant by centrifugation. After applying the required number of layers, the carbonate kernels were dissolved in a 0.2 M EDTA solution for 12 h. The resulting capsules were washed three times with water to remove core decay products. The microcapsules were obtained with an average diameter of 4.5 ± 1 μm. The size and number of microcapsules were measured using the dynamic light scattering method on a Zetasizer nano ZS device (Malvern, UK).

### 3.4. Measurement of Buffering Capacity

A suspension of microcapsules (PSS/PAH)_3_ (6.6 × 10^9^ microcapsules in 8 mL of water) was titrated with acid or base solutions in the pH range from 4 to 9. Titration was done by manual measurement using a Hanna pH 211 pH meter. The acid or alkali (with a concentration of 0.001 M or 0.005 M) was added to the solution to change the pH of the solution by 0.02 or more. Buffering capacity was calculated from Equation (1) after estimating the slope of the titration curves at each point by the variation of the pH between previous and subsequent injections [32]:

(1) $$BC=\frac{C_{acid\;or\;alkali}V_{acid\;or\;alkali}\;}{V_{s}\left(pH\left(i+1\right)-pH\left(i-1\right)\right)}$$

*BC*—buffer capacity
*C_acid or alkali_*—concentration of HCl of NaOH
*V_acid or alkali_*—volume of HCl of NaOH
*V_s_*—volume of solution
*i*—number of titration

## 4. Conclusions

In the course of this work, we studied the behavior of the buffer capacity of polyelectrolyte microcapsules depending on the concentration of capsules and the number of PMC shell layers. As a result, it was found that the buffering capacity of the entire suspension increases with increasing concentrations of microcapsules. However, at the same time, the calculated buffer capacity per 1 billion capsules in the pH range from 4 to 5.5 decreases with increasing concentration of PMC. This effect may be associated with an increase in the charge density of the capsule shell (the available -NH_2_ groups decreased) at high PMC concentrations. In addition, it was found that the main contribution to the buffer capacity of PMC is made by the entire shell of the microcapsule and not just its surface. At the same time, the buffer capacity of the capsules has non-linear growth with an increase in the number of PMC shell layers. This is presumably associated either with a decrease in the polyelectrolyte layer with an increase in their number or with a decrease in the permeability of hydrogen protons.

## Data Availability

Not applicable.
